# Aggressive Hydration With Ringer's Lactate in the Prevention of Post-ERCP Pancreatitis: A Meta-Analysis

**DOI:** 10.7759/cureus.14897

**Published:** 2021-05-07

**Authors:** Samar Aljohani, Hyder Mirghani

**Affiliations:** 1 Family Medicine, University of Tabuk, Tabuk, SAU; 2 Internal Medicine, University of Tabuk, Tabuk, SAU

**Keywords:** ringer's lactate, endoscopic retrograde cholangiopancreatography (ercp), post-ercp pancreatitis (pep), aggressive hydration

## Abstract

Post-endoscopic retrograde cholangiopancreatography (ERCP) pancreatitis (PEP) is a dangerous complication and occurs in a considerable number of patients. However, since well-randomized controlled trials investigating aggressive hydration with Ringer's lactate are lacking, this meta-analysis assessed the role of aggressive hydration with Ringer's lactate alone or in combination with other therapies in the prevention of PEP.

We searched PubMed, Cochrane Library, and Google Scholar for relevant articles. The search engine was set to randomize controlled trials and prospective cohorts assessing Ringer's lactate in PEP prevention either alone or in combination with non-steroidal anti-inflammatory drugs (NSAIDs) and stent. The keywords "aggressive hydration," "Ringer's lactate," "post-ERCP pancreatitis," "NSAIDs," "stent placement," and "somatostatin analogs" were used. The search was limited to a study on humans published in English with no limitation to the study period.

Two hundred and six articles were retrieved. Only eight articles fulfilled the inclusion criteria. The studies showed a reduction of post-ERCP pancreatitis using aggressive hydration with Ringer's lactate alone (odds ratio 0.23, 95% CI* *0.13 - 0.40, P-value < 0.001, I^2^ for heterogeneity = 0%, P-value = 0.61, Chi-square value 1.83, and degrees of freedom (df) 3. In addition, the combination of Ringer's lactate with stents or NSAIDs was superior to Ringer's lactate alone (odds ratio 0.63, 95% CI* *0.41 - 0.98, P-value < 0.04,* *I^2^ for heterogeneity = 0%, P-value = 0.48, Chi-square value 2.47, and df 3). Aggressive hydration with Ringer's lactate alone was effective in the prevention of PEP with a superior effect when combined with stents and NSAIDs.

## Introduction and background

Endoscopic retrograde cholangiopancreatography (ERCP) is an effective diagnostic and therapeutic procedure in the field of endoscopy. Post-ERCP pancreatitis (PEP) is a serious, stressful, and morbid consequence. PEP ranges from 2% - 40%, depending on the risk factors. Non-steroidal anti-inflammatory drugs (NSAIDs) have been shown to prevent PEP when administered intrarectally [1-2]. Aggressive hydration with Ringer's lactate for the prevention of post-ERCP pancreatitis is controversial [2]. Two possible mechanisms explain the anti-inflammatory effects of Ringer's lactate. Firstly, the lactate is metabolized to bicarbonate in the liver, resulting in low acidity. The second mechanism is the prevention of nuclear factor kappa B transcription factor inflammatory effects [3]. The topic of PEP is rapidly evolving. A recent study by Maruyama et al. concluded a large pancreatic volume was significantly associated with a higher incidence of PEP [4]. In addition, NSAIDs were shown to act selectively on cyclooxygenase 2 with no effect on cyclooxygenase 1 [5-6]. An important issue is the cost concerns. Regarding the cost, stents are costly, and the long duration of administering the lactated Ringer's also prolongs the recovery time, making the procedure less cost-effective (some high-volume centers have adopted a shorter time of administration) [7]. Thus, an update is highly needed. Few researchers have compared aggressive hydration alone or in combination with NSAIDs in the prevention of post-ERCP pancreatitis. Therefore, the current review aimed to assess the role of aggressive hydration with Ringer's lactate in the prevention of post-ERCP pancreatitis. We will also discuss whether the combination with NSAIDs is superior.

## Review

Eligibility criteria according to participants, intervention, comparison, outcome, and study design (PICOS)

We approached studies if they were randomized or cohort studies and compared the effects of Ringer's lactate alone or in combination with other post-ERCP pancreatitis preventive interventions. Only studies conducted among adult humans and published in the English languages were eligible. Animal and experimental studies, case reports, and case series were not included. The studies were included if they compared aggressive hydration with Ringer's lactate to standard hydration or comparing the same with aggressive hydration, plus NSAIDs and stent placement.

Interventions and outcome measures

Intervention

Intervention measures included 1) aggressive hydration with Ringer's lactate alone or in combination with other post-ERCP pancreatitis prevention methods, 2) aggressive hydration with Ringer's lactate is defined as 3 mL/kg/hour solution during ERCP and continued for eight hours afterward, and 3) the addition of a bolus dose of 20 mL/kg post-procedure [7].

Outcome Measure

The outcome is the development of PEP.

Searching methods and eligibility criteria

**Figure 1:**
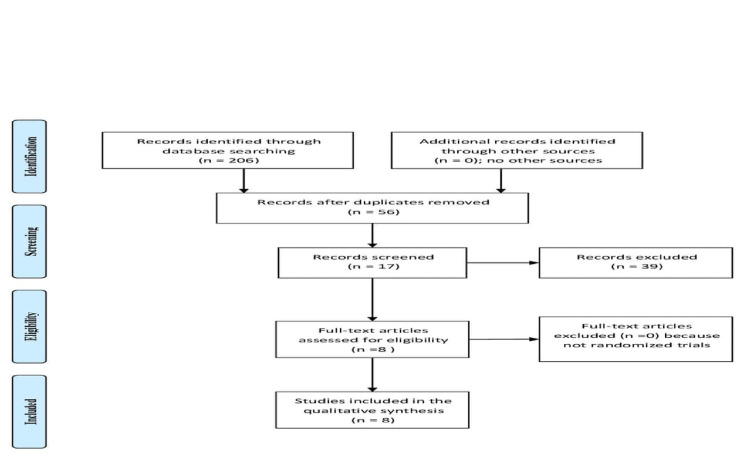
Aggressive hydration with ringer lactate for the prevention of post-ERCP pancreatitis ERCP: endoscopic retrograde cholangiopancreatography

A systematic electronic search was conducted in PubMed and Google Scholar for relevant articles in English. The search was limited to randomized controlled trials and cohort studies on humans from the first published article in 2014 to February 2021. Two researchers (HM and SA) independently searched the literature. Titles, abstracts, and references were screened for eligibility. Discrepancies were solved by a consensus between the two authors. The terms "Ringer's lactate," "aggressive hydration," "post-ERCP pancreatitis (PEP)," and "NSAIDs" were used with the protean "and". Articles published in languages other than English and studies on animals were not included. Of the 206 articles retrieved, 17 full texts were assessed and only eight articles fulfilled the inclusion and exclusion criteria. We used a modified Cochrane tool for the quality and risk of bias assessment [8]. The different phases of the search process are shown in Figure 1 and Table 1.

**Table 1:** Aggressive Hydration with Ringer's Lactate in the Prevention of Post-ERCP Pancreatitis ERCP: endoscopic retrograde cholangiopancreatography; RCT: randomized controlled trial

Author	Year	Country	Methods	Patients vs. placebo	Results
Buxbaum et al. [2]	2014	USA	RCT	0/39 vs. 4/23	Ringer's lactate was effective; 0% vs. 17%, P = 0.016
Ghaderi et al. [9]	2019	Iran	RCT	7/120 vs. 19/120	Ringer's lactate perioperative was effective; 5.83 vs. 15.83%, P = 0.013
Park et al. [10]	2018	South Korea	RCT	4/132 vs. 15/129	Aggressive Ringer's lactate was the best hydration method compared to standard; P = 0.008
Shaygan-Nejad et al. [11]	2015	Iran	RCT	4/75 vs. 19/75	Ringer's lactate was effective; P = 0.002
del Olmo Martínez et al. [12]	2020	Spain	A mixed cohort	7/227 vs. 18/414	No benefit of combination with diclofenac; P = 0.501
Hajalikhani et al. [13]	2018	Iran	RCT	1/107 vs. 3/112	Diclofenac combination with Ringer's not superior; P = 0.622
Mok et al. [14]	2017	USA	RCT	3/48 vs. 10/48	Ringer's lactate and indomethacin not superior to Ringer's, but reduced hospital stay; 6% vs. 21%, P = 0.04
Sotoudehmanesh et al. [15]	2019	Iran	RCT	26/207 vs. 33/207	Non-inferiority of a combination of a stent, pharmacological therapy, and Ringer's; 12.6% vs. 15.9%, P = 0.59

Statistical analysis

We used the last version of RevMan software, version 5.4 (Cochrane, London, UK). The included titles and data were entered manually; the fixed effect was used unless a significant heterogeneity necessitated a random effect (> 50%). A P-value of < 0.05 was considered significant.

Results

**Figure 2:**
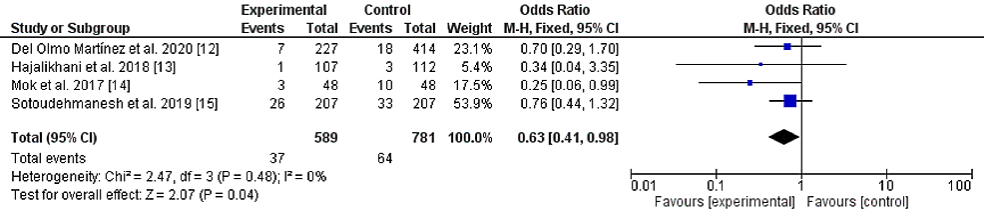
Ringer's lactate effects on post-ERCP pancreatitis CI: confidence interval; ERCP: endoscopic retrograde cholangiopancreatography; M-H: Mantel-Haenszel

**Figure 3:**
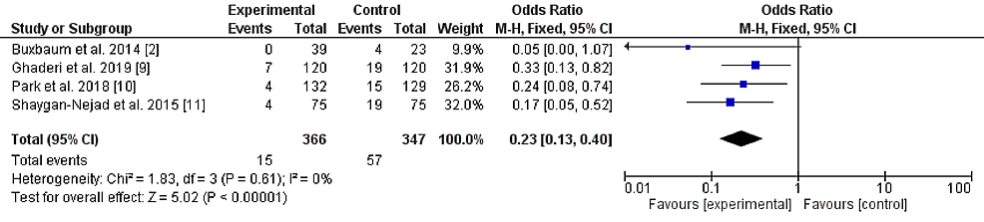
. Ringer's lactate in combination with other therapies effects on post-ERCP pancreatitis CI: confidence interval; ERCP: endoscopic retrograde cholangiopancreatography; M-H: Mantel-Haenszel

There were eight randomized controlled trials (five were from Asia [9-11, 13, 15], two from the USA [2, 14], and one from Europe [12]) including 713 patients who received Ringer's lactate infusion alone [12-15] or in combination with stenting or NSAIDs [2, 9-11]. All the studies showed that Ringer's lactate infusion was effective in the prevention of PEP. A highly significant statistical difference was found between Ringer's lactate and placebo regarding the prevention of PEP (odds ratio (OR) 0.23, 95% CI 0.13 - 0.40, P-value < 0.001, I^2^ for heterogeneity = 0%, P-value = 0.61, Chi-square value 1.83, degrees of freedom (df) 3) (Figure 2). Besides, a significant statistical difference was found between Ringer's lactate alone or in combination with other therapies regarding the prevention of post-ERCP pancreatitis (OR 0.63, 95% CI 0.41 - 0.98, P-value < 0.04, I^2^ for heterogeneity = 0%, P-value = 0.48, Chi-square value 2.47, df 3) [12-15]. The results imply that the combination is superior (Figure 3).

Discussion

In the present review, all the four RCTs investigating the effect of Ringer's lactate alone in the prevention of post-ERCP pancreatitis showed that the procedure is effective with a significant statistical difference (odds ratio 0.23, 95% CI 0.13 - 0.40, P-value < 0.001) [12-15]. A meta-analysis conducted by Wu et al. showed that Ringer's lactate aggressive hydration shortened hospital stay and reduced moderate to severe post-ERCP pancreatitis [16]. However, Wu and colleagues included only three randomized trials in their review. Zhang et al. reported similar results in their study, including only four trials (one unpublished) and three abstracts [17]. Radadiya and colleagues reviewed the literature and concluded that aggressive hydration with Ringer's lactate reduced post-ERCP pancreatitis, shortened the hospital stay by one day, and reduced hypernatremia with no increased adverse events [18].

Regarding the combination of Ringer's lactate with indomethacin or stent, four studies showed the superiority of the combination versus aggressive rehydration with Ringer's lactate (odds ratio 0.63, 95% CI 0.41 - 0.98, P-value < 0.04) [2, 9-11]. To our best knowledge, this is the first meta-analysis to assess the combination of aggressive hydration in combination with other therapy for the prevention of post-ERCP pancreatitis. Aggressive dehydration is defined as 3 mL/kg/hour lactated Ringer's solution during the procedure and for an additional eight hours afterward, as well as a 20 mL/kg bolus after the procedure, as compared with standard hydration at 1.5 mL/kg/hour during the procedure and for eight hours afterward. In addition, it might be not cost-effective in combination with stents, another costly and time-consuming procedure [19]. Previous studies showed the effectiveness of NSAIDs in the prevention of post-ERCP pancreatitis; the drugs were found to be safe in high-risk and unselected patients [20-22], and both diclofenac and indomethacin were shown to have equal weight either before or immediately after ERCP [23]. Rectal indomethacin, stent placement, and bolus administration of somatostatin appear to be most effective in preventing PEP [24]. Lactated Ringer's alone was effective and its effectiveness increased when combined with indomethacin or stent placement. However, the cost is a limitation. Also, extreme caution is needed not to increase the median blood pressure above 20 mmHg from the baseline due to a paradoxical effect (higher PEP) [19].

## Conclusions

Aggressive hydration with Ringer's lactate is an effective measure for PEP. The combination of lactated Ringer's with stents and indomethacin/diclofenac showed superiority. Further larger studies assessing the preventive interventions of PEP are recommended.
